# Modification of Dietary Fibers to Valorize the By-Products of Cereal, Fruit and Vegetable Industry—A Review on Treatment Methods

**DOI:** 10.3390/plants11243466

**Published:** 2022-12-10

**Authors:** Shahab Iqbal, Özge Tirpanalan-Staben, Knut Franke

**Affiliations:** Institute of Food Science and Human Nutrition, Leibniz University Hannover, 30167 Hannover, Germany

**Keywords:** dietary fibers, soluble dietary fibers, modification methods, by-product valorization

## Abstract

Many by-products originating from cereal, fruit and vegetable industries contain quite high amounts of dietary fiber (DF), which play an important role in maintaining the healthy state of the human body. Nevertheless, huge proportions of these by-products are still underutilized as feed ingredients, to generate energy within an anaerobic digestion plant or even landfilled. One of the biggest hindrances in the valorization of such by-products is their very low soluble dietary fiber (SDF) to insoluble dietary fiber (IDF) ratios, impairing their nutritional functionality, palatability and technological applicability. Therefore, it is of interest to develop methods that can enhance the SDF to IDF ratio and that can be applied to the by-product streams of the food industry, enabling better valorization perspectives for human nutrition purposes. In this regard, the review paper provides an overview of existing technologies to modify the SDF to IDF ratio in by-products of the food industry by means of physical, chemical and biological treatments. For each type of treatment, available data on application examples including achieved increases in SDF contents are given. Additionally, a comparative discussion regarding the advantages and disadvantages of these methods is provided.

## 1. Introduction

Dietary fibers (DFs) are part of the plant material, structural or complex carbohydrates that are resistant to breaking down in the human gastric track, as the human body lacks the enzymes necessary to digest these fibers. By being utilized mainly in the gastrointestinal (GI) track of the human body, DF’s are associated with several important physiological effects in the human body. These include maintaining a healthy GI track, reducing blood cholesterol levels, especially low-density lipoproteins, lowering blood glucose level and decreasing the risk of heart diseases, obesity and hypertension [1]. Additionally, there is an inverse relationship between DF consumption and the occurrence of certain types of cancer, as claimed by several studies [2].

With respect to their solubility, DF´s can be classified into water-soluble dietary fibers (SDFs) and insoluble dietary fibers (IDFs). Principally, SDFs can be broken down into smaller molecules by GI bacteria in the gut. This ability, also called fermentability, directly affects microbial diversity and function within the GI tract. Fermentation of SDF in the large bowel results in the generation of short chain fatty acids, which are associated with a number of beneficial effects including anti-inflammatory and antiproliferative [2,3]. On the other hand, the ability of gut microbes to ferment IDF is rather limited due to recalcitrance of this class of substances.

In addition to fermentability, the ability of DFs to thicken solutions and suspensions, signified as viscosity, is important for their physiological effects too. Several soluble polysaccharides, such as pectin, β-glucans, arabinoxylan, fructooligosaccharides and typical SDFs, can increase the viscosity of digesta and thereby trigger a prolonged gastric emptying, delayed nutrient release and slower transit through the small intestine. Therefore, the intake of such DFs is linked with decreasing glucose and cholesterol concentrations in the blood, and is connected to improving glycemic control as well as regulating serum cholesterol [4,5].

Therefore, SDF can be considered the healthier fraction of DFs with respect to their effects in the human body after being orally ingested. However, as will be discussed, most of the plant material, which could be a potential source of DFs, contains more IDFs than SDFs, limiting their positive health effects. Therefore, it is worth noting opportunities to transform IDFs into SDFs to increase their potential in improving health. This review will provide an overview regarding possible processes to increase the portion of SDFs, considering different types of treatments based on their fundamental principle: physical, chemical or biological.

DF contents in by-products from food manufacturing are naturally high, and yet are mostly underutilized. The focus of this review is directed toward by-products originating from the processing of cereals, fruits and vegetables. Although modifying the DFs in food and food by-products has been scientifically investigated in several studies, a comprehensive review regarding their application for by-product utilization as a food ingredient has not been published so far. Garcia-Amezquita et al. (2018) reviewed the processing of by-products from fruits and vegetables, and mainly focused on obtaining DF concentrates with special functionality [6]. Another review published by Yang et al. (2017) regarding the modification and application of dietary fiber, including the change of IDF to SDF, primarily focused on food without considering their by-products [7]. Therefore, this review provides new insights into the opportunities by which to valorize by-products of food manufacturing by enhancing their DF quality. In addition to the description of the relevant processes, molecular aspects of DF modification to increase solubility, as well as the pros and cons of treatment methods, will be discussed.

## 2. Sources of DF

Plants that are abundantly present in nature are the only source of DFs. Especially, fruits, vegetables, cereals and legumes can deliver a significant amount of DFs. Although rich sources of DF are present all over the world, their consumption is comparatively low and mostly does not cover the reference daily intake (RDI); i.e., 38 g/d for adult males and 25 g/d for adult females, with approximately 25% provided by SDF [3]. Most of the DFs sources contain significantly higher proportions of IDFs than SDFs [6].

Although many DF sources, such as vegetables, fruits and grains, provide sufficient amounts of DFs [3], industrially processing these products, mainly to increase their palatability, frequently results in significant losses in their DF content. For example, many industrial fruit and vegetable preparations require the separation of skin and pulp. Similarly, a relatively high proportion of cereal products require splitting and dehusking grains. These applications do not only decrease the DF content in the final products, but also generate enormous quantities of by-product streams that are rich in DF, albeit mainly dominated by IDFs.

Table 1 presents soluble and insoluble portions of DFs in several by-product streams of the vegetable, fruit and cereal industry. For comparison, Table 2 shows soluble and insoluble portions of DFs in several fruits, vegetables, legumes and cereal grains.

Table 3 demonstrates the amount of by-products of some fruits and vegetables, which sums up to an annual by-product accumulation of approximately 280 Mt considering only these specific fruits and vegetables [22]. Moreover, it is worth noting that many of the by-product streams from the fruit and vegetable industry are not only rich in DF, but also commonly demonstrate high antioxidant levels, mainly driven by their high polyphenol contents [23]; which is another reason for their utilization.

## 3. Methods to Convert IDFs into SDFs

Treatments to convert IDF into SDF can be classified into physical, chemical and biological methods. Mainly, mechanical forces are applied with or without the application of heat in physical methods. In chemical methods, reagents such as acid and alkali are used to break down bonds in complex IDFs to form SDFs. Biological methods apply purified enzymes or specific microorganisms, producing the required enzymes to break down the complex structure of IDF and convert them into SDF.

### 3.1. Physical Methods

Table 4 summarizes the effect of different physical treatments on the SDF content of the by-products.

#### 3.1.1. High Hydrostatic Pressure

High hydrostatic pressure (HHP) is a non-thermal physical treatment that has the potential to increase SDF content by breaking covalent and non-covalent bonds within fiber-related carbohydrates [29]. Effects of HHP treatment on foodstuffs are mainly based on two principles: the Le Chatelier principle and the isostatic principle. The Le Chatelier principle relies on the fact that the application of pressure favors all reactions that are connected to decrease the molecular volume. The isostatic principle means that pressure is equally applied in all parts of the foodstuff, irrespective of its shape and size.

The HHP treatment of soybean by-product, at 400 MPa and 60 °C, increased the SDF content from 2.1% to 19.7%, thereby increasing the SDF to IDF ratio by eight-fold. Surprisingly, researchers have also reported a 42% increase in total DF content after HHP application [31]. Degradation of DF was investigated in orange peel by means of HHP process applied at 600 MPa at 55 °C. The results revealed an increase of up to 95% in SDF content, and a corresponding decrease in IDF content could also be observed [30]. However, it must be noted that the application of HHP did not produce consistent results regarding the level of increase in the SDF content depending on the processing parameters, as well as the compositional details of the applied food. While the HHP treatment of prickly pear peel conducted at 22 °C increased SDF content by 22%, the same process conducted at 55 °C induced an increase of less than 2% in SDF content. The reduction in the yield of SDF at the higher temperature could be attributed to a higher degree of hydrolysis of the DF, resulting in smaller molecules, which are no longer effective as DF. Interestingly, and comparable to the results of the previously mentioned study conducted with soybean by-product, the authors observed an increase in the total DF and IDF contents after HHP application on prickly pear peel [29]. A shift from soluble to insoluble fibers mainly occurs in polysaccharides containing uronic acids, arabinose and galactose. According to the authors, two endogenous enzymes, pectin methylesterase (PME) and polygalacturonase (PG), were responsible for lowering the solubility of DF. PME demethoxylates the pectin in the prickly pear peels and produces free carboxyl groups that are readily available to interact with divalent ions. In this way, a cross-linked structure of the pectic chains is developed, decreasing solubility. On the other hand, PG hydrolyses glycosidic linkages in the pectin molecule, decreasing the polymerization degree and viscosity of the polysaccharides. Therefore, changes in the solubility of DFs result from the superimposition of both reactions [39].

Alterations in the molecular structure of DF do not only influence SDF content, but also contribute to the water retention and water swelling, and fat absorption capacities, as well as the α-amylase activity inhibition ratio and bile acid retardation index, which was reportedly observed in de-oiled cumin DF as a result of HHP treatment [40]. This effect was observed for both the single use of HHP and in combination with enzyme treatment.

#### 3.1.2. Ultrasonic Waves

Ultrasonic waves (UW) can be applied to food matrices with sufficient liquid content, through which the UW are conveyed. Even though data regarding the alteration in the SDF to IDF ratio by means of UW treatment is scarce, the effect of ultrasonic power for the purpose of dietary fiber extraction from by-product streams of fiber-rich products has been widely studied [41,42]. The ultrasonic assisted extraction of SDF from apple pomace conducted at pH 2.0 and 80 °C with a solid-liquid ratio of 1:20 resulted in a SDF yield of 16.4% after 40 min of sonication at 400 W, which was higher in comparison to microwave-assisted extraction (14.9%) and conventional acid extraction (10.3%). However, this is lower in comparison to enzymatic extraction of SDF from apple pomace with cellulase applied at a concentration of 0.5% based on apple pomace (18.7%) [43].

On the other hand, the UW-assisted extraction applied to coffee silver skin, a by-product of coffee fruit processing, at 200 W for 20 min resulted in an extraction yield of 22.8%, which was less than the yields obtained by only microwave-assisted SDF extraction (37.2%) and conventional solvent extraction (29.4%) [44]. The authors explained that the higher SDF extraction yield with a faster increase in internal temperature and pressure was due to the microwave effects.

Chen et al. (2011) studied the effect of temperature and time on the UW-assisted extraction of SDF from carob pods. They reported a maximum extraction yield of 6.7% was achieved with treatment at 700 W and 60 °C for 45 min. Beyond these limits, the extraction efficiency started to decrease, meaning that the SDF contents became lower [45].

#### 3.1.3. Thermal Treatments

Thermal treatments such as sterilization, steam processing, boiling, frying, roasting and pressure-cooking are able to break the glyosidic linkages in DF, resulting in the formation of simpler carbohydrates with shorter chain lengths and less branched structures. Thermal treatments are frequently applied for varied purposes where DF modification is not necessarily the main goal. Therefore, modification of DF can be considered as a side effect during such processes. Benítez et al. (2011) studied the influence of heat treatment at a temperature of 115 °C for 17 to 31 min on onion by-products and found a reduction of IDF of up to 31%. Thereby, the ratio of soluble-to-insoluble fiber in these by-products increased from 1:3 to 1:2 [33].

Autoclaving of wheat bran for 1.5 h at 121 °C decreased SDF content from 3.1% to 2.9%, and increased it from 2.3% to 2.8% in rice bran [32]. The reason behind the decrease in SDF was attributed to the removal of other substances during the washing steps, which reduced the overall solubility of the sample and increased IDF content.

#### 3.1.4. Extrusion

Extrusion as a food processing technique is mainly used in the production of a wide variety of snack foods, breakfast cereals and pet foods. Many factors influence the chemical reactions occurring during extrusion, such as moisture content, temperature, residence time and screw speed (shearing). Nevertheless, heat is the most important factor along with pressure and shear forces in modifying the texture of plant tissue and reorganizing the fiber components [46]. High temperatures and shear forces induce the breakdown of long macromolecular chains producing smaller molecules with higher solubility, thereby increasing SDF content [47].

Extrusion experiments, carried out at a relatively low temperature of 115 °C, a feed moisture of 31% and a screw speed of 180 rpm, were able to increase SDF content from 2.05% to 12.6% in a soybean by-product. In addition to increasing SDF content, these treatments also increased water and oil retention capacity, as well as swelling capacity, of DF in the residue [37]. A study investigating the effects of a twin-screw extrusion-cooking process on the modification of DF in garlic skin, with a feed moisture content of 25%, an extrusion temperature of 170 °C and a screw speed of 170 rpm, reported a trebling of SDF content, which was initially 5.3% [38].

A twin-screw extrusion-cooking process to modify wheat bran, using a feed moisture content of 6.1–17.5%, a feed rate of 20–32 kg/h, an extrusion temperature of 100 °C and a screw speed of 240–250 rpm, delivered an 8 to 16% increase in SDF content [34]. An even higher increase in SDF content was reached after extrusion of raw pea hulls, applying feed moistures of up to 60%, feed rates of up to 20 kg/h, temperatures of 100 °C and screw speeds of up to 250 rpm. In this case, SDF content rose from 4.1% before extrusion to 13.1% after extrusion [35]. Using extrusion to modify the DFs in apple pomace with a relatively low feed moisture of 22%, a high screw speed of 700 rpm and a moderate temperature of 100 °C, resulted in an increase of SDF of only about 34% (from 12.5 to 16.7%) [36].

### 3.2. Chemical Methods

Chemical methods use the addition of acid and/or alkaline substances to shift the pH value resulting in the solubilization of DF. The main factors involved in this treatment are the amount of acid and alkaline used, temperature and reaction time. Particularly, the consecutive acid-alkaline treatment seems to be a promising method for increasing SDF content. Here, the initial acid treatment increases the surface porosity of the fiber particles, making them available for the hydroxyl groups to penetrate. During the subsequent alkaline treatment at higher pH values, hydrolyzation occurs more effectively [48]. Scientific studies investigating chemical methods regarding their modification capacity of DF fractions, specifically from by-products of agri-food resources, are quite limited. Therefore, Table 5 summarizes the effect of chemical treatment methods on the SDF content of both by-products and of food materials primarily high in DF.

Feng et al. (2017) used 15% alkaline hydrogen peroxide at a pH value of 11 and a solid-to-liquid ratio of 1:18 (*w*/*v*) to treat black bean coats for 0.5 h and found an increase in the level of SDF from 7.8% to 16.9% [49]. According to the authors, alkaline hydrogen peroxide treatment could reduce the crystallinity of cellulose and could rupture the hydrogen bonding in molecular chains, opening internal structures in fibers and increasing the SDF content in products. Bader Ul Ain et al. (2018) used different chemical treatments (acid treatment, alkali treatment, acid-alkali treatment and alkali-acid treatment) and found that the acid-alkali treatment (6.0 N HCl and 6.0 N NaOH) for 1 to 4 h was most effective method in increasing the SDF content of barley fiber, where a rise of up to 7.7 times was recorded [52]. However, it should be noted that, despite the effects of the acids and bases, long-term heating at 90 °C might contribute to the increase in SDF content as well.

In another study, two varieties of wheat and sorghum were treated in the same way and an increase in SDF content was reported with factors of 7.4 and 8.7 in wheat and sorghum varieties, respectively [48].

A doubling of SDF content was reached after long-term treatment (24 h) of ground wheat bran with a particle size of 90 µm using an acetate buffer (pH 4.8) at 55 °C [50]. An increase of only around 14% in SDF content was observed after chemical treatment of black bean coats with 2% sulfuric acid at 100 °C for 30 min, followed by a treatment with sodium hydroxide as the alkaline [51].

### 3.3. Biological Methods

Although biological treatments are frequently applied in food processing, research on the application of these methods in order to modify the DF and to increase the SDF content is scarce. Biological methods utilize enzymes to meet specific goals in the modification of DF. Purified enzymes can be either directly applied or they can be utilized within a process using appropriate species of microbes, the latter being termed as fermentation. Two types of fermentation, submerged and solid-state fermentation, can be distinguished. In a submerged fermentation, a high moisture content of more than 90% is used and microbes grow in the liquid medium. On the other hand, a lower moisture content, typically between 30 to 80%, is applied in solid-state fermentation and microbes grow on a moist solid substrate. Both technologies have been determined to modify the DF and increase the amount of SDF. Table 6 summarizes the available data regarding the effect of biological treatment methods on SDF content of several high DF containing foods and by-products. Some data are represented based on the extraction efficiency of SDF. It should be noted that the majority of studies reporting certain improvements have resulted from a fermentation process applied after a preceding heat treatment process (autoclaving), which contributes to the alterations in DF content and properties.

#### 3.3.1. Enzymatic Treatments

Enzymes used in DF modification to increase the SDF content are mainly xylanase, cellulase and lignin oxidase [7]. An increase in SDF content could be achieved by the hydrolysis of insoluble cellulose and hemicellulose to obtain soluble cell wall polysaccharides after an enzymatic treatment [60], which principally requires a preceding pretreatment process to increase the availability of the DF for the enzymatic access.

One study showed that using Ultraflo L^®^ (enzyme blend of beta-glucanase and xylanase) to hydrolyze the previously autoclaved soybean by-product resulted in a DF content with improved physicochemical properties, as well as higher SDF content [61]. In another study, 5% (*w*/*v*) Viscozyme L solution, an enzyme blend of beta-glucanases, pectinases, hemicellulases and xylanases, was added to tomato peels mixed with acid-sodium citrate buffer solution using a solid-to-liquid ratio of 1:10 (*w*/*v*) and a pH value of 6. Compared to the untreated tomato peels, the enzymatic treatment conducted at 45 °C for 35 min resulted in a 72.3% increase in the extraction yield of SDF [53].

#### 3.3.2. Submerged Fermentation

Fermentation uses organic acids and enzymes naturally produced by microorganisms, which aid in the degradation of cellulose and hemicellulose, thereby forming more porous and looser structures of polysaccharides. The reactions reduce the degree of polymerization in cellulose, hemicellulose and lignin, resulting in a significant increase in SDF content, as well as some improvements in DF properties, such as increased water, oil and swelling capacities [55]. Fermentation of millet bran using Bacillus natto at an incubation temperature of 37 °C for 47 h enhanced the SDF content from 2.3% to 13.2% [55]. Similarly, fermentation of defatted rice bran by Trichoderma viride at 28 °C for 72 h achieved an increase in SDF content from 10.5% to 33.4% [54].

#### 3.3.3. Solid State Fermentation

Solid-state fermentation (SSF) is a common technique for the production of microbial metabolites by utilizing different agricultural wastes, such as rice straw, sugarcane bagasse, wheat straw, rice hulls and corn cobs. SSF results in high product concentration with relatively low energy being required. The water added in SSF is absorbed in a solid matrix by the substrate, facilitating the growth of microorganism by means of sufficient transfer of oxygen. Molds and bacillus species are mostly applied in SSF to modify DF and improve antioxidant capacity, because they can produce a series of cell wall hydrolases and proteases. The weak acidic environment generated by the enzymatic actions can disintegrate the dense network of cell wall components of IDF origin and result in an increase in SDF content [62].

By tuning the SSF parameters, such as particle size, moisture content, temperature, lighting and media composition, the DF content of the substrate can be influenced [56,57]. Wu et al. (2012) treated sweet potato residues with an SSF process by applying *Schizophyllum commune*, a fungal species of Basidomycota, with the parameters of 1.8 to 2.5 mm particle size, 65% moisture content, 27 °C temperature and natural lighting. They reported an increase in the extraction yield of DF and SDF from 22% to 70% and from 3% to 17%, respectively [56]. In another study, wheat bran was treated with *Hericium erinaceus*, an edible higher fungal species also belonging to the Basidomycota division, in SSF at 28 °C for 6 days. Soybean meal as a nitrogen source was added along with KH2PO4, ZnSO4, FeSO4 and MgCl2 to aid in SDF production. The results revealed that a 4.68-fold increase in SDF was achieved compared to wheat bran without fermentation [57].

Zhao et al. (2017) tried another approach by which to modify DF in wheat bran, starting by autoclaving it at 120 °C for 20 min and then fermented it either with yeast, lactic acid bacteria or both yeast and lactic acid bacteria at 37 °C for 24 h with 50% moisture content. The highest increase in SDF content, from 4.43% to 8.36%, was found in wheat bran fermented with only lactic acid bacteria. Fermentations using only yeast or the combination of both types of microbes were less successful [58]. Similarly, Mao et al. (2020) applied lactic acid bacteria Enterococcus faecalis in a SSF with sterilized wheat bran at 37 °C for 36 h. They measured an increase in SDF content from 1.2 to 4.1% [59].

### 3.4. Comparison of Advantages and Disadvantages of the Modification Methods

Physical modification methods can be regarded as easy to perform, as they do not involve the addition of chemicals, which usually generate waste or demand resources required for their recycling. Additionally, compared to chemical and biological modification methods, physical methods require short reaction times [63].

Concerning the extrusion process, one of the most important advantages is the very short time required to achieve the modifications. Other advantages of extrusion include low operation cost, high productivity, versatility, unique product shapes and energy savings [37]. Moreover, losses of vitamins and amino acids are comparatively low in extrusion due to the short retention time of the material in the extruder.

On the other hand, long-term exposure to high temperatures during heat treatment could undesirably modify the techno-functionality of DF and result in low quality DF [33,41]. Moreover, the high-energy consumption of some physical processes could reduce cost effectiveness. In this respect, HHP treatment can be seen as a high-efficiency and environmentally friendly method for improving DF quality [40].

Compared to the biological methods, chemical modification methods can be also considered as time-efficient. Depending on the chemical agents used, SDF content can be efficiently increased, combined with relatively low damage to the molecular structure of the DF [48]. However, exposure to severe conditions, i.e., corrosive chemicals in high concentrations with or without the application of high temperatures, can lead to undesirable modifications negatively affecting the quality of the DF [41]. Moreover, the use of strong acid and alkali agents in chemical treatments may create significant amounts of waste with negative effects for the environment.

Biological methods that include enzymatic treatment and microbial fermentation are considered as environmentally friendly. They work in relatively mild conditions with respect to pH and temperature values [7,62]. Due to the specificity of the enzymes involved, these methods result in comparatively low destruction of the DF structure. Furthermore, the methods do not result in chemical pollution and they are well established in food processing. Fermentation can be regarded as a simple and inexpensive processing technique to achieve desirable changes in the raw material composition and to improve palatability [64]. On the other hand, biological methods can be cost-intensive should they require the addition of purified enzymes. Moreover, due to the mild conditions kept during biological processes, sterilization of the substrate prior to fermentation is commonly required in order to avoid the growth of undesired microorganisms that naturally occur in the substrate. Furthermore, considering the SSF process, it can be difficult to control fermentation conditions, since an automated process control system is difficult to apply for large-scale SSF processes [56,59]. Finally, and importantly, biological modification processes require at least many hours, and often days, for the growth of microbes, the secretion of enzymes and the modification of the substrate, making these methods time and resource intensive.

Although biological treatments are frequently applied in food processing, research on the application of these methods in order to modify the DF and to increase the SDF content in by-products is rather scarce and should be expanded to increase the utilization potential of such products. 

### 3.5. Combination of Different Types of Methods

One of the strategies by which to minimize the disadvantages of single treatment methods, as well as to maximize the desired effect of these applications, is the combination of different treatment methods. Several studies investigating combined treatments have reported greater effect on the transformation of IDF into SDF than the use of a single method. Although a combination of treatment processes always leads to higher costs in equipment and prolongs treatment time, sometimes it is the only way to reach the desired level of DF transformation. The available literature regarding studies conducted on the combination of methods to increase SDF content in by-products of food processing is rather limited. Therefore, some examples of the combined treatment of food are included in the following chapter, to demonstrate some of the general effects, which are comparable to those in by-products.

Bader Ul Ain et al. (2019) examined the effect of chemical treatments both alone and combined with extrusion process. SDF content increased from 3.0% to 4.5% in acid-treated wheat samples and it could be further increased by up to 6.0% by a following extrusion treatment. Interestingly, the SDF content decreased from 9.9% in alkaline-treated wheat samples to 6.1% in alkaline-treated-extruded samples. The same effect was observed in Sorghum, where SDF increased from 1.7% to 3.1% after acid-treatment and further increased to 6.1% in the acid-treated-extruded samples. However, the SDF content decreased to 6.2% after subjecting an alkaline-treated sample to a subsequent extrusion treatment, whereas an SDF content of 11.3% was directly obtained after the alkaline-treatment [48].

On the other hand, investigations of the effect of pressure-cooking alone and in combination with acid and alkaline treatment on the modification of barley dietary fiber revealed a higher increase in SDF content, from 7.2% to 16.8%, in the samples after the alkaline treatment compared to pressure-cooking alone [52]. Similarly, the application of ultrasonic-assisted alkaline treatment was also tested on the residues of the microalgae *Nannochloropsis oceanica* obtained after lipid extraction. Such a treatment revealed a 150% increase in the extraction efficiency compared to the alkaline treatment alone [65].

In another study, Song et al. (2018) started with a pre-treatment of bamboo shoots in an extruder followed by an enzymatic treatment using cellulase at a pH value of 4.5 and a temperature of 50 °C for 4 h. While the extrusion only increased the SDF content from 4.2% to 10.4%, the enzymatic treatment of previously extruded samples led to an SDF content of 22.2% [60]. Interestingly, one study reported no significant improvement in SDF content following a HHP treatment of de-oiled cumin fiber. Nevertheless, a following enzymatic (laccase and cellulase) treatment was able to increase the SDF content from 12.2% to 30.4% [40].

A positive effect of the combination of extrusion and a following either acid or alkaline treatment compared to the application of a single method was observed by Ning et al. (1991) [66]. However, the best results were found for acid treatment followed by alkali treatment. The additional influence of extrusion before or after this combination was not investigated by the authors.

## 4. Conclusions

The modification of DF in order to increase its SDF content is of interest, especially for high DF containing industrial by-product streams of the food industry. Such an upgrade in nutritional and technological functionality would allow improved valorization strategies of these by-product streams, which are otherwise mostly underutilized, e.g., as substrates for anaerobic digestion.

Several treatments based on physical, chemical and biological methods are able to increase the amount of SDF. Their extent depends on the type of method, matrix and treatment conditions. However, it is not possible to identify a general method with certain conditions that is similarly successful in all food matrices. The efficiency of the conversion of IDF into SDF is affected by several factors, such as the type of carbohydrate as well as other components of the food matrix, possible side reactions and process parameters. Assuming the interdependency of most of these factors, some unexpected results might be observed. One example is a decrease in SDF content with a concomitant increase in IDF content due to some polymerization reactions.

Nevertheless, one promising way to increase the effectiveness of the conversion of IDF into SDF is the suitable combination of different types of methods, e.g., physical and biological treatments. A sufficient understanding of the reaction pathways for specific food systems is required to choose the optimum combination with respect to the order of treatments and their parameters.

Finally, further studies are required regarding the functionality and applicability of by-product streams with modified DF content in different food preparations.

## Figures and Tables

**Table 1:** Dietary fiber contents of by-product from processing of several fruits, crops and cereals (DF, SDF and IDF contents are given in g/100 g).

Foodstuff Origin	DF	SDF	IDF	Portion of SDF on Total DF Content in %	Reference(s)
**Fruits**					
Banana peel	50.6	6.1	44.5	12	[6]
Carob Pods (without seeds)	40.4	6.8	33.6	17	[8]
Grape pomace	62.4	4.3	58.1	7	[9]
Mamey sapote peel	58.1	7.3	50.8	13	[6]
Orange peel	57	9	47.6	16	[6,10]
Prickly pear peel	38.1	7.0	31.1	18	[6]
Tamarind Bagasse	63.5	3.9	59.6	6	[6]
Watermelon peel	41.5	3.2	38.3	8	[6]
Maca residues	35.0	3.3	34.7	9	[11]
**Root and tuber crops**					
Carrot peel	45.5	10.4	33.6	23	[6,12]
Onion peel	62.1	7.4	54.7	12	[6,13]
Potato peel	50.5	8.7	41.8	17	[6,14]
Red beetroot peel	26.6	6.9	33.5	21	[15]
**Cereals**					
Oat bran	19.5	5	14.5	26	[6,16]
Oat bran	19.5	5	14.5	26	[6,16]
Wheat bran	52.2	3.8	48.4	7	[6,16]

**Table 2:** Dietary fiber contents of several fruits, vegetables, root and tuber crops, legumes and cereals (DF, SDF and IDF contents are given in g/100 g).

Foodstuff	DF	SDF	IDF	Portion of SDF on Total DF Content in %	Reference(s)
**Fruits**					
Apple	2.0–2.2	0.2–0.7	1.5–1.9	10–30	[17,18]
Avocado	5.5	2.0	3.5	37	[18]
Banana,	1.7- 1.8	0.5–0.6	1.2	29–32	[17,18]
Guava,	12.7	1.5	11.8	12	[18]
Mango	1.8	0.7	1.1	41	[17]
Orange	1.6	0.3	1.3	19	[19]
Plum	2.2	0.6	1.7	25	[19]
**Vegetables**					
Bitter gourd	16.6	3.1	13.5	19	[20]
Broccoli	3.3–3.5	0.3 0.4	3.0–3.1	9–13	[17,18]
Cauliflower	1.8	0.7	1.1	39	[17]
Cucumber	1.1	0.2	0.9	18	[18]
Spinach	2.6	0.5	2.1	19	[17]
**Root and tuber crops**					
Beet root	7.8	2.4	5.4	31	[20]
Carrot	2.5–5.7	0.2–1.6	2.3–4.1	8–28	[17,18,20]
Onion	1.9	0.7	1.2	37	[18]
Potato	3.2	0.6	2.6	19	[20]
**Legumes**					
Green peas	3.5	0.3	3.2	9	[17]
Lentils	11.4	1.1	10.3	10	[17]
White Beans	17.7	4.3	13.4	24	[17]
**Cereals**					
Oats	10.3	3.8	6.5	37	[17]
Rice	9.9	4.4	5.4	44	[21]
Sorghum	12.2	2.7	9.5	22	[21]
Wheat	12.4–12.6	2.3–3.5	9.0–10.2	18–28	[17,21]

**Table 3:** Worldwide annual production volumes of some fruits and vegetables in 2020, and the concentration of their non-edible portion (%) as potential by-product streams (Mt: million tons), (Source for production: [22]).

Food	Production	Non-Edible Portion (%)	Reference (for Non-Edible Portion Only)
Apples	86.4	25	[24]
Bananas	119.8	35	[25]
Grapes	78.0	20	[25]
Mangoes, mangosteens, guavas	54.8	40–60	[26]
Onions, dry	104.6	17–38	[25]
Oranges	75.5	30–50	[27]
Papayas	13.9	47	[28]
Peas, green	19.9	40	[25]
Pineapples	27.8	30	[25]
Potatoes	359.1	15	[25]
Tomatoes	186.8	20	[25]

**Table 4:** Some examples of physical treatment methods applied to influence the DF composition (SDF content is related to initial dry mass, values marked by * are related to total initial mass).

Treatment Type	Material	Time (min)	Temperature (°C)	Pressure (MPa)	SDF Contents and Change (%)			Reference
					Initial	Final	Change **	
High hydrostatic pressures	Mango peel	10	55	600	15.2	17.5	15	[29]
	Orange peel	20	55	600	7.2	14.0	95	[30]
	Prickly pear peel	10	22	600	8.5	10.3	22	[29]
	Prickly pear peel	10	55	600	8.5	8.6	2	[29]
	Soybean by-product	15	60	400	2.1	19.7	845	[31]
Thermal	Wheat bran	90	121	0.2	3.1	2.9	−7	[32]
	Rice bran	90	121	0.2	2.3	2.8	21	[32]
	Onion by-product	17–31	115	n.m. *	8.8	10.5	19	[33]
Extrusion	Wheat bran	n.m.	120	n.m.	8.1	up to 16.0	up to 98	[34]
	Raw pea hulls	n.m.	100	n.m.	4.1	up to 13.1	220	[35]
	Apple pomace	n.m.	100	n.m.	12.5	16.7	34	[36]
	Soybean by-product	n.m.	115	n.m.	2.1 *	12.6 *	514	[37]
	Garlic skin	n.m.	170	n.m.	5.3 *	15.9 *	199	[38]

* n.m. not mentioned by the authors, ** Change = (SDF content Final − SDF content Initial)/SDF content Initial 100%.

**Table 5:** Some examples of chemical treatment methods applied to influence DF composition. (SDF content is related to total initial mass).

Material	Chemical Agent & Concentration (%)	Temperature (°C)	Reaction Time (min)	SDF Contents and Change (%)			References
				Initial	Final	Change *	
Black bean coats	Alkaline hydrogen peroxide (15%)	-	30	7.8	16.9	117	[49]
Wheat bran (particle size 90 µm)	Acetate buffer at pH 4.8	55	1440	7.9	16.3	106	[50]
Black bean coats	2% H_2_SO_4_ 1.5% KOH	100	30 (acid)	4.4	5.2	14	[51]
Barley	6 N HCl 6 N NaOH	90	60–240	4.7	41.2	771	[52]
Wheat	6 N HCl 6 N NaOH	90	60–240	3.1	26.5	768	[48]

* Change = (SDF content Final − SDF content Initial)/SDF content Initial · 100%.

**Table 6:** Some examples of biological treatment methods applied to influence the DF composition. (SDF content is related to total initial mass, values marked by * are related to total dry mass).

Treatment	Material	Enzyme/Microorganism Applied	Temperature (°C)	Reaction Time (h)	SDF Contents and Change (%)			References
					Initial	Final	Change *	
Enzymatic Treatments	Tomato peel	Viscozyme L	45	0.6	8.9	15.4	72	[53]
Submerged fermentation	Defatted rice bran	*Trichoderma viride*	28	72	10.5	33.4	218	[54]
	Millet Bran	*Bacillus natto*	37	47	2.3	13.2	474	[55]
Solid state fermentation	Sweet potato residue	*Schizophyllum commune*	27	672	3	17	467	[56]
	Wheat bran	*Hericium erinaceus*	28	144	2.7	13.1	385	[57]
	Wheat bran	Lactic acid bacteria (LAB)	37	24	4.4	8.4	89	[58]
	Wheat bran	*Enterococcus faecalis*	37	36	1.2	4.1	245	[59]

* Change = (SDF content Final − SDF content Initial)/SDF content Initial · 100%.
